# The Treatment of Cysts

**Published:** 1890-09-27

**Authors:** 


### THE TREATMENT OF CYSTS.

Dr. Edward Woakes, senior aural surgeon to the London Hospital, has recently published an article in the *Lancet* on the treatment of cystic goitre, ranula, and other cysts with chromic acid. Some years back he had under treatment a woman for fibro-cystic goitre, involving the right lobe of the thyroid body. The cyst, which extended to within an inch of the angle of the jaw, had been tapped, and injected with concentrated solution of tannin, with the result of securing apparent obliteration of the cavity; but after some months the cyst refilled, the line of tapping becoming a sinus. Dr. Woakes adopted the plan of passing a probe, previously dipped for three inches of its length into a saturated solution of chromic acid. This was done several times in three successive weeks, when, after an interval of non-attendance, Dr. Woakes was surprised to see a brown tough material protruding from the wound. Traction brought out a rolled-up mass, which when spread out was about the size of the palm of the hand, and was undoubtedly the wall of the cyst. After this deliverance, the cystic part of the trouble was ended. Shortly afterwards another case occurred in a female. Dr. Woakes had not at this time realised sufficient confidence in the use of chromic acid, so tannin injection was used. But the cyst was simply changed into an abscess. Chromic acid was then introduced through an "acid carrier" (an instrument which holds a definite quantity of the fluid in its distal end, and enables the operator to apply the acid directly to the boundaries of the cavity). The patient was directed to keep her bed, and to apply ice if any trouble followed. Nothing however occurred, and at the end of a week adhesion was complete, and there has been no return of the cyst since. In a third case there existed a fibro-cystic enlargement in the left lobe of the thyroid. This was treated solely with chromic acid. The cyst disappeared, and the wound healed rapidly. The fourth case mentioned was that of a male, æt. 30. The right lobe of the thyroid was dense and fibrous, whilst a small, but prominent, cyst occupied the middle line. It was tapped, and about six drachms of clear straw-coloured fluid escaped. The acid was then applied as in the preceding case. There were absolutely no symptoms, and at the end of a week no trace of the cyst could be detected. After an interval of a month a cyst was tapped in the right lobe. About two drachms of fluid escaped, and there was rather free bleeding. On the cessation of the bleeding the acid was introduced. The wound healed at once, but the cavity refilled very rapidly, owing, as Dr. Woakes thinks, to the acid having been neutralised by the blood remaining in the cavity before the acid reached the buundaries. The operation was repeated after a few weeks, and was quite successful.

A young man presented with a large ranula which occupied the right side of the floor of the mouth, nearly filling the mouth, and further projected below the jaw on this side as a large elastic swelling the size of a hen's egg. The tumour had already been submitted to unsuccessful treatment by excision of mucous membrane. A small incision was made in the most projecting portion of the tumour in the mouth, and through it the thick gelatinous contents were carefully evacuated. There was no bleeding. Explored with a blunt probe the cavity was found to extend under the tongue almost to the opposite jaw. The wound gaped freely, and allowed the "carrier," charged with solution of chromic acid, to be applied to its inner surface. The patient was put to bed, a precaution which again proved unnecessary, and no sign of irritation followed. At the end of a week there was no trace of swelling or enlargement, but a probe could be introduced for a short distance into the wound, all beyond this point being firmly adherent. Acid was applied to this sinus, after which it rapidly healed. Dr. Woakes remarks that the precise action of chromic acid on the walls of cysts is not known. He thinks repeated observation points to the complete and rapid amalgamation between the tissues and the acid, to such an extent, that thereafter neither the one nor the other retains its former physical character. Now, chromic acid is a powerful oxidising agent, decomposing alcohol, glycerine, etc. It has been used as a caustic, and a watery solution—1 in 4 or stronger—to warts, condylomata, and lupus. A solution of 1 in 40 is also a good application to ulcerated gums, and especially to syphilitic affections of the tongue or pharynx. If we recollect right chromic acid was first used as an escharotic by Mr. John Marshall, but we do not remember that it has previously been used as an injection after the method of Dr. Woakes. Tapping and injection of iodine has been generally used for cystic goitre. Sir Morell Mackenzie recommended a watery solution of perchloride of iron, 25 per cent. As regards the treatment of ranula there may be a calculus present, and if so, removal of this formation is a necessity, for while it remains no kind of treatment will be effectual. An injection of solution of chloride of zinc, 45 grains to the ℥i, has been much recommended by Panas and others. Also injections of iodine or ergotin. Dr. Woakes seems to have great faith in the chromic acid injection for the cure of cysts, and the several successful cases detailed justify such confidence, and demand further trial by other surgeons. Dr. Woakes does not bring forward one successful case of ranula as establishing the efficacy of the mode of treatment, but he opines there is sufficient cause shown for extended trial.

